# Cost effectiveness of pre-referral antimalarial treatment in severe malaria among children in sub-Saharan Africa

**DOI:** 10.1186/s12962-017-0076-5

**Published:** 2017-07-14

**Authors:** Vivian Rakuomi, Faith Okalebo, Stanley Ndwigah, Levi Mbugua

**Affiliations:** 1 Ministry of Health, Nairobi, Kenya; 2 School of Pharmacy, University of Nairobi, Nairobi, Kenya; 3 School of Mathematics, The Technical University of Kenya, Nairobi, Kenya

**Keywords:** Cost-effectiveness, Decision analysis, Modeling, Disability Adjusted Life Years

## Abstract

**Background:**

In 2013, 78% of malaria deaths occurred in children aged 5 years and below, in sub-Saharan Africa. Treatment of severe malaria requires a health facility with inpatient care. However, in most sub-Sahara African countries, access to health facilities is a major problem. Pre-referral antimalarial treatments aim to delay the progress of severe malaria as patients seek to access health facilities. Rectal artesunate can be administered in the community as a pre-referral treatment in rural hard-to-reach areas. In Kenya, though pre-referral rectal artesunate has been included in the National Guidelines for pre-referral treatment, it is yet to be implemented in the public healthcare system. It is important, therefore, to establish its cost-utility compared to current parenteral treatments. This study evaluated the cost-utility of provision of pre-referral treatments by community health workers compared to similar services at a primary health facility.

**Methods:**

This was a decision model-based cost-utility analysis, comparing pre-referral antimalarial treatments provided by: community health workers (CHWs), primary health facility, direct access to a tertiary health facility and no access to treatment. A theoretical cohort, of 1000 children, who were below 5 years old; residing in rural hard-to-reach areas, was taken as the reference population. Data was collected through key informant interviews, to assess the costs, while key measures of effectiveness, were obtained from existing studies. The key measure of outcomes was Disability Adjusted Life Years (DALYS) averted. Probabilistic sensitivity analysis was carried out to assess the robustness of the model.

**Results:**

Provision of rectal pre-referral treatment by community health workers was estimated to avert 13,276 DALYs, at a cost of $68,428 for a cohort of 1000 children. Provision of rectal pre-referral treatment at a primary health facility was estimated to avert 9993 DALYs, at a cost of $73,826 for a cohort of 1000 children, while going directly to a tertiary health facility was estimated to avert 15,801 DALYs, at a cost of $114,903 for a cohort of 1000 children. The incremental cost effectiveness ratios for provision of pre-referral treatment by community health care and primary health workers were $5.11 and $7.30 per DALYs averted respectively.

**Conclusion:**

Use of CHWs was more cost effective than provision of pre-referral treatments at a primary health facility especially, with high referral compliance. Rectal artesunate can easily be administered by community health workers, unlike parenteral pre-referral interventions.

**Electronic supplementary material:**

The online version of this article (doi:10.1186/s12962-017-0076-5) contains supplementary material, which is available to authorized users.

## Background

Malaria is a vector-borne infectious disease of the red blood cells caused by a parasitic protozoan of the Plasmodium genus. Severe malaria is characterized by vital organ dysfunction and it is therefore a medical emergency that can result in death [1]. Therefore, correct diagnosis and early initiation of treatment are very important in reducing mortality and complications [2]. However, not all children who are febrile are taken to hospital and therefore, they do not receive any treatment [3].

In Kenya, poor health infrastructure, especially in the rural areas, delays prompt access to health services, leading to increased mortality from malaria [4–6]. This has led to concerted efforts to increase access to treatment, including the adoption of rectal artesunate as a pre-referral intervention. Rectal artesunate may offer advantages over parenteral artesunate and parenteral quinine, since it can be administered at home by care-givers or community health workers (CHWs). In pediatric patients, rectal artesunate has a better safety profile with regards to side effects compared to quinine [7]. It is also, more effective in inducing rapid parasite clearance within 24 h, hence reducing the risk of death by approximately 49% [8–11]. Studies have shown that the utilization of community-based management increased the possibility of accessing antimalarial drugs as well as positively impacting on treatment-seeking behavior by almost 90% in some areas in Africa [12, 13]. In Kenya CHWs are trained according to policy guidelines on the astute use of antimalarial pre-referral treatment. This policy requires prior testing using rapid diagnostic test (RDTs) kits before initiation of antimalarial therapy [14].

Although rectal artesunate has been adopted into the Kenyan National Malaria Guidelines, it is yet to be implemented. Given that funding for malaria does not meet the burden experienced, it was vital to assess whether pre-referral treatment options, as currently practiced, are cost-effective compared to rectal artesunate. This study therefore, evaluated the cost-utility of pre-referral malaria treatments in Kenya, provided by CHWs, versus primary health facility. The population of study was children less than 5 years, living in rural hard-to-reach areas, with high malaria endemicity.

## Methods

The study design was a prospective model based cost-utility analysis, using decision analytic modeling. The study focused on a theoretical cohort of 1000 children, under the age of 5 years, residing in rural areas, with high malaria endemicity [4]. Children aged less than 5 years, comprise 17.4% of the population in this region. The proportion of males and females in this age-group was 50.6 and 49.4% respectively [15].

The study was carried out from the perspective of the Government of Kenya, as the largest provider of healthcare services in Kenya [16]. The time frame for the intervention was 5 years, which was the time from birth to the age of 5 years. The main strategy of interest was provision of rectal pre-referral treatment by CHWs.

There were four comparator groups; provision of rectal artesunate by CHWs, the use of a primary health facility without any inpatient services, direct access to a tertiary facility, and no access to any form of treatment as the no intervention arm. These groups were selected to represent the various levels of healthcare services provided in Kenya. The main outcomes of interest were Disability Adjusted Life Years (DALYs), which were computed from the expected mortalities, and neurological sequelae post malarial treatment. Incremental cost effectiveness ratios, and cost effectiveness ratios, were computed using the DALYs, and costs associated with each of the strategies.

### Measure of effectiveness of pre-referral treatments in childhood malaria

DALYs were computed as the sum of Years of Life Lost due to premature mortality (YLL) and Year of Life Lived with Disability (YLD) [17]. Years of life lived with disability were computed as the product of duration of the morbidity or complications, incidence and the disability weights [18]. A cohort of children with signs of severe malaria, aged between 0 to 5 years, was assumed to receive treatment from a community health worker, primary healthcare or a tertiary healthcare facility. In line with the Kenyan policy for management of malaria, the children were assumed to have tested positive using the rapid diagnostic kit. The fourth cohort received no treatment. The standard life expectancies of male and female children aged 0–1 and 1–4 years was obtained from the WHO life tables [19]. The average age at death was assumed to be 0.5 and 2.5 years for the age group less than 1 and 1–5 years respectively. The number of deaths due to severe inpatient malaria was estimated, using a case fatality rate of 7.5% (3.5–9.3%), as obtained from a Kenyan study [20]. The case fatality rate for untreated malaria was estimated at 70%. This was obtained from a study that sought expert opinion on case fatality rate for untreated febrile illnesses [21]. This is because no clinical study has been done in this population, and these estimates are usually subjective. The life expectancy for children aged 0–1 years, was 63.1 years for males and 65.6 years for females; and for children aged 1–4 years, was 65 for males, and 67.5 for females, as obtained from WHO life expectancy ranking, for Kenya [19]. The disability weights were obtained from WHO data for the sub-Saharan region. From this data, the disability weight associated with a malaria episode was 0.211, neurological sequelae was 0.4710, and anemia was 0.013 [17]. Table 1 summarizes the variables used to compute effectiveness.

**Table 1:** Epidemiological parameters used in calculating Disability Adjusted Life Years

Epidemiological prevalence and effectiveness	Point estimate (%)
Inpatient case fatality rate of malaria	7.5
Case fatality rate of untreated malaria	70.0
Average length of in-hospital stay	5.0
Effectiveness of rectal artesunate	49.0
Probability of neurological sequelae	3.0
Probability of anemia	18
Assumed effectiveness of i.m quinine vs rectal	90
Assumed effectiveness of i.m artesunate vs rectal	120
Life expectancy	
Males (0–1 year)	63.1
Females (0–1 year)	65.6
Males (1–4 year)	65
Females (1–4 year)	67.5
Disability weights	
Malaria episode	0.211
Neurological sequelae	0.471
Anemia	0.013

The incidences of neurological sequelae, and anemia, were 3 and 18% respectively, for those seeking treatment, as obtained from literature [20, 22]. The length of hospitalization, was a median of 5 (3–8) days [22]. The duration of neurological sequelae, was estimated to be 2 years, while anemia complications were estimated to last for about a month [23–25].

Administration of rectal artesunate followed by inpatient care has been shown to reduce mortality due to severe malaria by 49% (95% CI 19.31–67.76) [8]. There was no study comparing effects of pre-referral rectal artesunate, i.m artesunate and i.m quinine on mortality. This study therefore, assumed that, effectiveness of i.m quinine, was 90%, that of rectal artesunate, with regard to reduction in mortality. This assumption was based on studies comparing the parasite clearance rate of rectal artesunate, versus i.m quinine [9, 11, 26]. The effectiveness of i.m artesunate, was assumed to be 120%, that of rectal artesunate, with regard to reduction in mortality. This assumption was made based on a study that showed the superiority of i.m artesunate, after 24 h of illness [9, 10, 26]. It was assumed that patients sought treatment within 24 h of onset of illness. Studies have shown that pre-referral treatments are more effective within this time [8, 9]. Those who received pre-referral treatment but did not seek inpatient care had the same risk of dying as those who did not seek any treatment [24].

The study assumed that the incidence of anemia in the population not seeking treatment was twice that of those who sought inpatient care after pre-referral treatment [4]. Children aged less than 1 year were assumed to comprise 20% of the cohort and 1–4 years 80% of the cohort. The female to male ratio was 1.024:1, according to the Kenya Population Census done in 2009. Years of Life Lost for each age group and sex was separated computed and summed up. The Year of Life Lost were age-discounted using a discount rate of −0.03.

### Costing methodology

Costs were obtained from literature and from key informant interviews. The key informant interviews were carried out in agencies involved in procurement, supplies, distribution and management of malaria in Kenya. These include National Malaria Control Program (NMCP), Kenya Medical Supplies Authority (KEMSA), African Medical Research Foundation (AMREF) and Clinton Health Access Initiative (CHAI).

Inpatient costs were estimated at $75.13 (36.33–102.64), per patient. Costs of managing neurological sequelae, were estimated at $48.52, and, malarial anemia costs were $45.02, as obtained, from studies done in Kenya [20, 27]. All the costs were updated to 2015 rates using the Kenya consumer price index and then converted to international dollars using the purchasing power parity for Kenya against the US dollars [28, 29]. Table 2 summarizes costs of input variables as obtained from key informant interviews.

**Table 2:** Program level costs obtained from key informant interviews

Item	Cost ($)
Implementation costs	
Training of trainers for case management (per person)	552.2
Training of healthcare workers (per person)	496.9
Training of CHWs (per person)	25.1
Monitoring and evaluation	96,625.4 (62,633–195,890.8)
Printing of guidelines (each)	12.4
Printing of manuals (each)	25.8
Acquisition costs	
I.m artesunate (per vial)	1.49–1.62
I.m quinine (per vial)	0.20
Rectal artesunate (50 mg)	0.105–0.350
Procurement	2%
Warehousing	3%
Distribution	5%
Personnel monthly salaries	
CHW	41.4–165.63
HCW (nurse)	1242.23–2070.39

*CHW* community healthcare worker, *HCW* healthcare worker

The cost of time a CHW spends treating a child was calculated using a 2-h workday, working for 5 days a week and a salary range of 41.4–165.63, as obtained from key informant interviews and from literature [30]. The cost of time a healthcare worker spends per child was calculated with an estimated working duration of 40 h per week. We assumed that every patient needed 20 min of care. The costs obtained were annuitized with a discount rate of 8% taking into account future capital costs over a period of 3 years [31].

Kenya Essential Medicines Supplies Authority (KEMSA) charges 2% of purchase price for tendering, however not all acquisitions are through KEMSA and therefore this cost does not apply to all the stocks of drugs warehoused and distributed by KEMSA. The capital costs were calculated per healthcare provider, providing services to one case of severe malaria. From a key informant interview, the number of CHWs in western Kenya was estimated at 7100.

The total yearly case load for malaria was 170,000, with an estimated prevalence of 10% for severe malaria [32]. We therefore estimated that each CHW treats 2.4 cases of severe malaria per year. From the 2008 Kenya National Human Resource for Health strategic plan, 22.7% of the entire health work force was working in the Western region. The total number of healthcare workers in Kenya, trained in malaria case management is estimated at 6000, as obtained from key informant interviews. This study therefore estimated that 1800 of the trained healthcare workers are from this region. Children less than 5 years of age were estimated, at a population of 1,687,787 in this region [15].

The yearly prevalence for malaria was 38% in this population, and 67.3% utilized Government facilities [4, 33, 34]. We estimated incidence of severe malaria of 10% among the population with malaria [32]. We therefore multiplied these rates with the population, then divided with the number of healthcare workers trained in malaria case management in the region, to get 24 cases treated per healthcare worker in a year. We ignored shared capital costs like training of trainers of trainers and printing of materials as they are shared by all the treatment arms, and costs of managing adverse reactions, as their prevalence is low.

### Decision analytic modeling

The decision analytical tree was drawn to reflect the treatment-seeking options accessible to a caregiver with a severely ill child in remote rural areas.

**Fig. 1:**
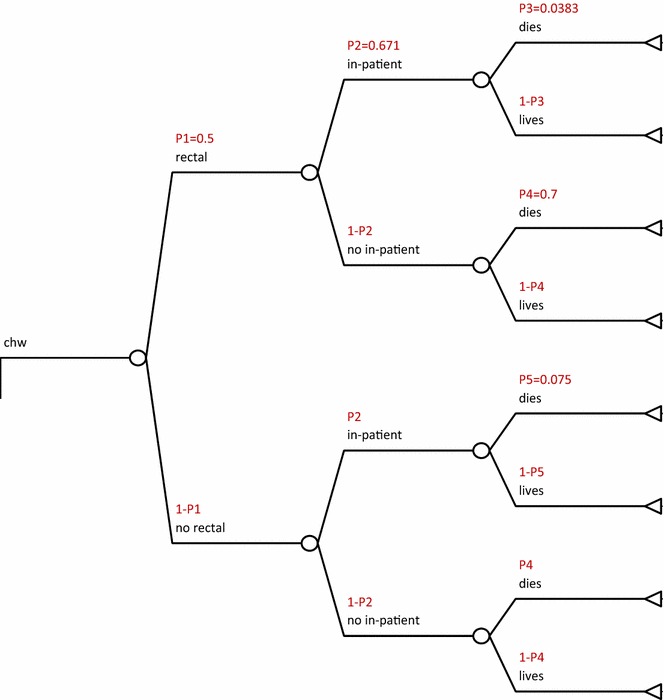
Cost utility of rectal artesunate provided by community health workers. *P1* intervention uptake, *P2* referral compliance, *P3* inpatient case fatality rate following rectal pre-referral, *P4* case fatality rate for untreated severe malaria, *P5* inpatient case fatality rate without any pre-referral

**Fig. 2:**
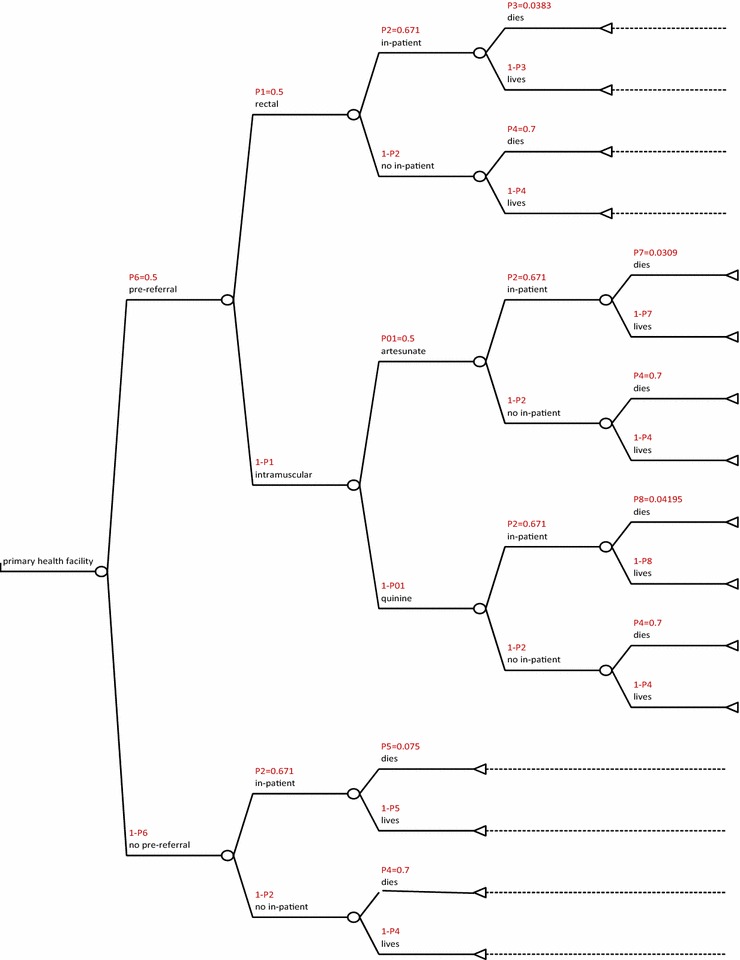
Cost-utility of pre-referral treatments at primary facility. *P1* probability of receiving any of the pre-referral interventions, *P01* probability of receiving either of the intramuscular interventions, *P2* referral compliance, *P3* inpatient case fatality rate after rectal pre-referral treatment, *P4* case fatality rate for untreated severe malaria, *P5* inpatient case fatality rate for severe malaria without pre-referral treatment, *P6* intervention uptake, *P7* inpatient case fatality rate with pre-referral i.m artesunate, *P8* inpatient case fatality rate with pre-referral i.m quinine

**Fig. 3:**
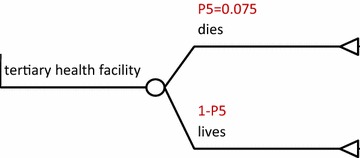
Cost-utility of a tertiary facility. *P5* inpatient case fatality rate for severe malaria (with no pre-referral interventions)

**Fig. 4:**
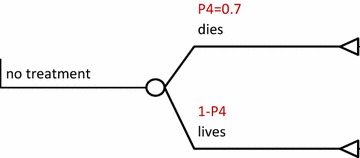
Cost-utility of not seeking treatment. *P4* case fatality rate for untreated severe malaria

The options were; a visit to a CHW then refer, a visit to a primary health facility then refer, directly seeking care at a tertiary health facility and not seeking any treatment. This is shown in Fig. 1: cost utility of rectal artesunate provided by community health workers, Fig. 3: cost-utility of a tertiary facility, and Fig. 4: cost-utility of not seeking treatment. The study assumed equal probability of receiving or not getting pre-referral treatment. At primary facility, patients would be put on rectal artesunate, i.m quinine, i.m artesunate or get no treatment as shown in Fig. 2: cost-utility of pre-referral treatments at primary facility. The study assumed that at the tertiary facility all severe malaria cases were admitted.

The following assumptions were made in the model; that treatment would be sought within 24 h of onset of severe illness and that a referred patient would get appropriate treatment within 24 h of the referral to maximize effectiveness of rectal artesunate [9, 11]. The study also assumed that pre-referral treatment would be given only once, at point of contact and thereafter, patients would be referred. Referral compliance was assumed to be similar, from either the CHW or primary health facility and was estimated at 67.1% [12].

### Sensitivity analysis

Multivariate probabilistic sensitivity analysis was done using non-Bayesian (frequentist) method approach. The input parameters that were varied are those that had the largest influence on one-way sensitivity analysis. The list of parameters that were investigated, their probability distribution functions and the arguments are presented in Table 3 (Additional file 1).

**Table 3:** Effectiveness and cost input variables used in the analysis

Probability	Distribution	Distribution parameters
Years of life lived	Gamma	Mode 28,732 (r = 5, λ = 0.00017)
Cost of salaries, PHF	Gamma	Mode 3450 (r = 5, λ = 0.00144)
Inpatient cost for a mortality	Gamma	Mode 49,420 (r = 5, λ = 0.00010)
Inpatient costs of survival	Gamma	Mode 123,550 (r = 5, λ = 0.00004)
YLD not admitted, survives	Gamma	Mode 33 (r = 5, λ = 0.14844)
YLD admitted and survives	Gamma	Mode 33 (r = 5, λ = 0.14923)
YLD admitted but dies	Gamma	Mode 1.70 (r = 5, λ = 2.88345)
No rectal artesunate	Beta	Mode 0.5 (α = 2, β = 2)
Compliance	Beta	Mode 0.67 (α = 2, β = 1.49000)
Inpatient CFR	Beta	Mode 0.075 (α = 2, β = 13.33333)
Cost of RDTs	Gamma	Mode 545 (r = 5, λ = 0.00917)
Efficacy of rectal	Beta	Mode 0.49 (α = 2, β = 2.04080)

For each parameter, a vector of 1000 randomly assigned values was generated and these were used to compute 1,000,000 values of ICER. The median and inter-quartile ranges were reported. Two-way sensitivity analyses on variables of clinical and economic importance were conducted. The analysis was done using Base R software version 3.2.2.

## Results

The results of the base model were obtained and presented in Table 4. From these results, the expected costs associated with no treatment of severe malaria were $0.88, for a cohort of 1000 patients with 20,122 DALYs. Provision of pre-referral treatment by CHWs, and in a primary health facility, would result in an aversion of 15,535 DALYs, and 12,610 DALYs respectively. The expected cost of pre-referral management of a cohort of 1000 children aged less than 5 years was much lower for CHWs, at $85,492. The same services provided in a primary health facility had a cost of $88,961. The most cost-effective option was pre-referral treatment by CHWs, followed by subsequent treatment at a tertiary healthcare facility. The incremental cost-effectiveness ratio (ICER) for this intervention was $5.50 per DALY averted. A direct visit to the tertiary health facility was more cost-effective (ICER $6.90 per DALY averted), as compared to pre-referral treatment provided in a primary health facility, with subsequent treatment at a tertiary health facility (ICER $7.05 per DALY averted).

**Table 4:** Costs incremental health outcomes and cost-utility of pre-referral antimalarial treatments by healthcare provider

Scenario	Intervention	Costs for a cohort of 1000 children ($)	DALYs	DALYs averted	Incremental cost ($)	ICER ($ per DALY averted)
Base model	No treatment	0.89	20,122	–	–	–
	CHW	85,491	4587	15,535	85,490	5.50
	PHF	88,961	7512	12,610	88,960	7.05
	THF	123,711	2186	17,936	123,710	6.90
Probabilistic sensitivity analysis	No treatment	0.88 [0.88–0.88]	19,529 [15,315, 24,737]	–	–	–
	CHW	68,428 [43,362, 100,703]	5413 [3091, 8430]	13,276 [9534, 17,684]	68,427 [43,361, 100,702]	5.11 [3.01, 8.21]
	PHF	73,825 [47,531, 103,875]	8328 [5030, 12,531]	9993 [6180, 14,429]	73,825 [47,530, 103,874]	7.30 [5.30, 11.20]
	THF	114,903 [91,842, 145,168]	3111 [1801, 5005]	15,801 [11,990, 20,375]	114,902 [91,841, 145,167]	7.14 [5.10, 10.92]

*CHW* community healthworker, *PHF* primary health facility, *THF* tertiary health facility, *ICER* incremental cost-utility ratio, *cost* cost for treating one child

**Fig. 5:**
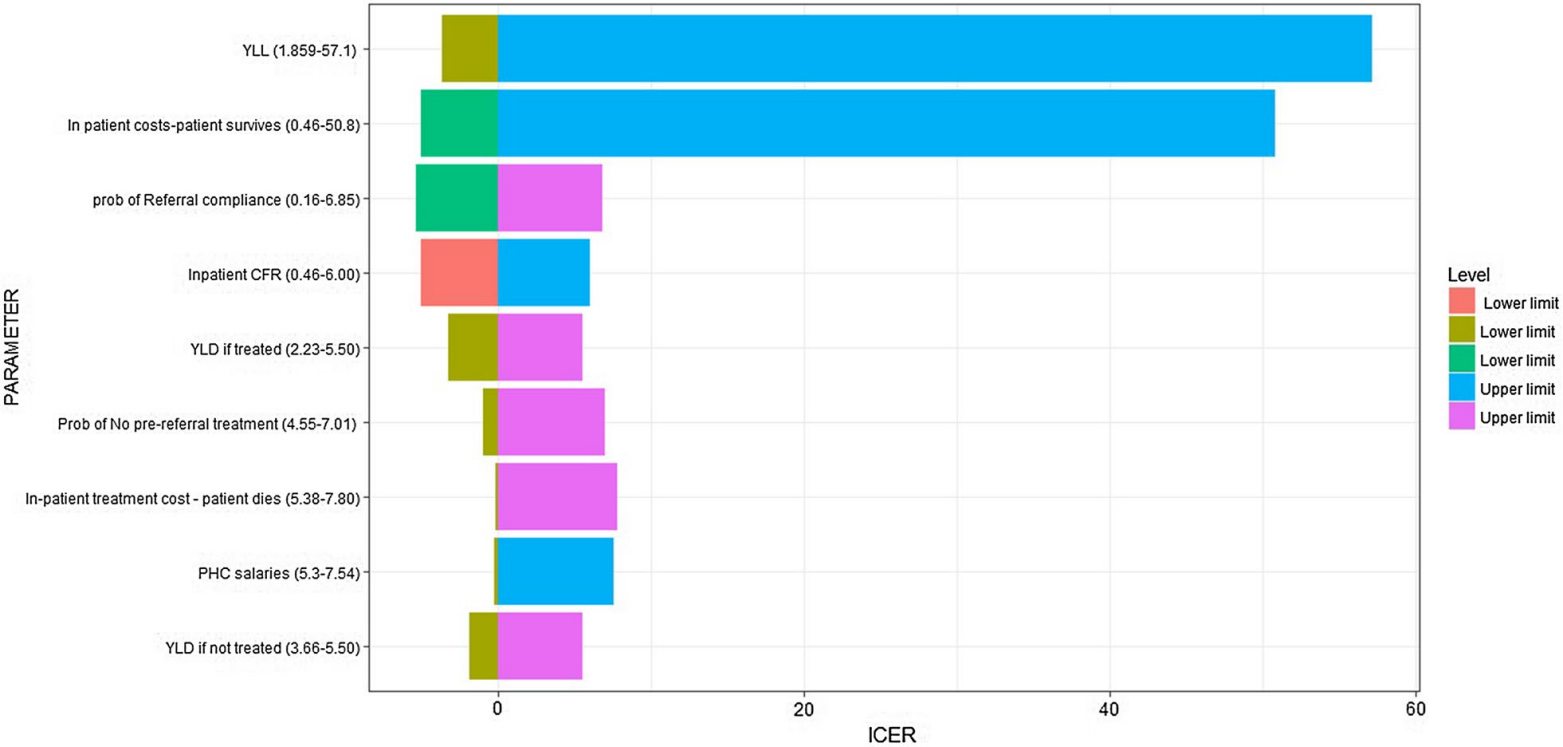
Effects of input variables on the cost utility of pre-referral treatments

One-way sensitivity analysis (Additional file 2) was conducted, and the results on the variation of ICER for the CHW intervention were summarized as a tornado diagram, in Fig. 5: effects of input variables on the cost utility of pre-referral treatments. For the sake of parsimony, variables that had no or minimal effect on the ICER were excluded from the tornado diagram. For all interventions, assumptions about Years of Life Lost (YLL), the cost of inpatient care at a tertiary health facility, and the inpatient case fatality rate had the greatest impact on the ICER.

Intramuscular quinine, rectal artesunate and intramuscular artesunate increased the incremental cost, but the overall effect on ICER was minimal. The cost of managing complications, anemia and neurological sequelae had no effect on the incremental costs and ICER. This was attributed to the fact that these costs are shared across all interventions. Similarly, the cost incurred by patients who do not seek treatment from the community health workers, primary and tertiary health facilities, had no effects on the ICER. The impact of referral compliance on ICER was not very pronounced.

The probability of receiving intramuscular quinine, versus intramuscular artesunate, was excluded from the tornado diagram. This parameter had almost no effect on both the incremental cost and the ICER. Assumptions around the relative efficacy of rectal artesunate, as opposed to that of i.m quinine and i.m artesunate had minimal effect on the ICER of service provision.

The results of multivariate probabilistic analysis confirmed earlier findings of the base model and are presented in Table 4. From these results, provision of pre-referral treatment by CHWs had the potential of averting 13,276 DALYs, at a cost of $68,428 for treating a cohort of 1000 children. The option of pre-referral treatment, at a primary health facility had the potential of 9993 DALYs, at a cost of $73,825 for treating a cohort of 1000 children. Going straight to a tertiary facility without pre-referral treatment, has the potential of averting 15,801 DALYs, at a cost of $114,903 for treating a cohort 1000 children. The most cost-effective option was the use of a CHW (ICER of $5.11 per DALYs averted), followed by direct access to a tertiary health facility (ICER of $7.14 per DALYs averted), and then primary health facility (ICER of $7.30 per DALYs averted). Receiving no form of treatment was the cheapest option, with a cost of $0.88 for a cohort of 1000 children. It was however, associated with the largest disease burden, of 19,529 DALYs (Additional file 3).

The incremental costs of a patient receiving services increased as one moved from CHW to PHF ($2429, ICER −1.45) (Additional file 4) and similarly from PHF to THF ($34,098, ICER $6.74).

On conducting two-way sensitivity analysis, we noted that increasing the probability of a patient receiving rectal artesunate from a CHW, reduced the point estimate of ICER (Additional file 5).

The ICER increased from a base case of $5.11 (IQR $3.01, $8.21), to $1.37 (IQR $0.82, $2.36) over a range of assumptions of the efficacy of rectal artesunate, in a severely ill child. If all children received rectal artesunate without a confirmatory diagnosis and these reduced the chances of inpatient mortality by 6%, the point estimate of ICER indicated that the intervention would still be beneficial.

## Discussion

The use of CHWs in the provision of pre-referral treatment was associated with fewer DALYs as well as more DALYs averted, as compared to seeking treatment at a primary health facility. It was also more cost effective and less costly. A recent study in low income countries suggest that the use of CHWs could be as cost-effective as primary healthcare workers due to tangible and non-tangible benefits offered by CHWs [35]. The results obtained were in agreement with other studies looking at the utilization of community healthcare workers [29, 36–38]. The use of CHWs is cost-effective where there is high uptake and utilization of community strategy as well as adherence to referral advice by care-givers. Enhanced supervision and strengthening of community strategy to avoid irrational prescribing will ensure that this option remains cost effective in rural remote areas with little access to healthcare facilities especially in relation to direct patient costs. However, the effect of irrational use did not reduce the cost effectiveness of community strategy, suggesting that even in cases where testing is unavailable, giving rectal artesunate to all febrile children had some benefit. The only cost of note was the acquisition cost of the pre-referral interventions (Additional file 6).

The results illustrate the difference in cost of seeking health services between different levels of healthcare categories. In this case, the study found the option of using CHWs and then going to a tertiary health facility more cost effective than going to primary health facility followed by a referral to a tertiary health facility. Utilization of CHWs first before referral was also more cost effective than going directly to a tertiary facility. This is expected, given no infrastructural investments of offering services at community level, as opposed to infrastructure costs as well as expertise, at primary and tertiary facilities. Therefore, given the long-term nature and cost of putting up healthcare infrastructure, the use of CHWs to offer pre-referral treatments remains an important option in mitigating the effects of severe malaria, in rural hard-to-reach areas (Additional file 7).

The difference in cost-utility between CHWs and primary health facility could also be explained by differences in the salaries, number of cases and hours of work of a CHWs and a healthcare worker, as well as transport costs and other attendant costs of seeking healthcare.

The finding of cost-utility analysis was sensitive to referral compliance, both with CHWs, and at primary healthcare facility though this was not very pronounced and remained the same across the two options. With increased referral compliance, DALYS averted increased since more patients are admitted for inpatient care. The effect of compliance on cost-utility, was similar to a study done on use of rectal pre-referral treatments [39]. This suggests that strategies to increase referral compliance, including closer support supervision of CHWs and CHW-assisted referral should be adopted. In cases where referral compliance is very low, however, CHWs may be empowered to give repeat doses of rectal artesunate, as this has been shown to be effective as a treatment alternative [40].

In the option where patients went directly to a tertiary health facility, they had better outcome with fewer DALYs and more DALYs averted than any other option. However, it was the most costly and this finding was in line with a costing study done in Kenya [25]. Seeking treatment directly at a health facility with inpatient services within 24 h, decreases the duration to initiation of treatment, and has better health outcomes [4, 41, 42]. Therefore, a tertiary health facility, even though costlier, gives the best health outcomes, and, should ideally be the first point of contact, for those with severe malaria. However, capital costs of putting up a tertiary facility in resource limited areas are prohibitive. This has lead to a situation where health facilities are far apart and have limited infrastructure to effectively handle most of the healthcare needs of the population. The other challenges experienced in these settings include few trained medical personnel. Therefore, the use of community healthcare workers, serves as a cost-effective measure, to address the gaps in healthcare resource allocation, and distribution, while ensuring better health outcomes.

## Conclusion

Malaria remains one of the leading causes of mortality in sub-Saharan Africa. The most vulnerable group to this disease, are children under the age of 5 years. Therefore, efforts should be put in place to roll back the impacts of malaria in this population. These efforts include the adoption of rectal artesunate and improving access and ease of use to those at risk. However, for rectal artesunate to be successfully implemented, local research data on cost effectiveness, as well as behavioral and cultural practices should be considered (Additional file 8).

The use of CHWs, in provision of rectal pre-referral treatment was a cost-effective option, when compared to the provision in a primary health facility. However, the benefits of CHWs would only be realized where there is a clear strategy to enhance provision and uptake of CHWs services. Full compliance to referral advice would be necessary as well. Coupled with the use of CHWs, a lot of sensitization on health-seeking habits in febrile illnesses would increase uptake of this intervention. Cultural beliefs, as concerns use of rectal route of administration, would have to be debunked and its safety highlighted.

This study recommends the strengthening of community strategy in regards to uptake and referral compliance. This should involve strict supervision and adherence to treatment guidelines, which require testing using rapid diagnostic tests (RDTs), before administration of any antimalarials to avoid irrational use. Given that malaria infections can occur more than once in a child less than 5 years, a cost utility study based on a Markov model could be done to determine effects of both pre-referral treatment, and curative applications of rectal artesunate. The study also recommends a further study on patient related costs, side effects, and delay to access treatment. In this study, the same case fatality rate was used for those who sought no treatment, and those who had pre-referral treatment, but did not seek inpatient treatment. It is probable that these two groups had different case fatality rates.

The results from this study can be used to persuade policy makers, to provide rectal artesunate as a pre-referral intervention in rural hard to reach areas.

## Additional files

**Additional file 1.** Code for the distributions.

**Additional file 2.** Cost one-way sensitivity analysis.

**Additional file 3.** DALYS modeling.

**Additional file 4.** Difference in ICER between CHW and PHF.

**Additional file 5.** Result PSA.

**Additional file 6.** Results for the base model.

**Additional file 7.** Two way sensitivity analysis.

**Additional file 8.** Decision tree model.

## Abbreviations

CHW: community health workers
PHF: primary health facility
THF: tertiary health facility
DALYs: Disability Adjusted Life Years
ICER: incremental cost effectiveness ratio
KEMSA: Kenya Medical Supplies Authority
CER: cost effectiveness ratio
RDT: rapid diagnostic test
WHO: World Health Organisation
YLD: Years Lived with Disability
YLL: Years of Life Lost
