# Supplementary material for: Cost effectiveness of pre-referral antimalarial treatment in severe malaria among children in sub-Saharan Africa
Source: Cost Eff Resour Alloc. 2017 Jul 14;15:14. doi: 10.1186/s12962-017-0076-5 (PMC5512821; doi:10.1186/s12962-017-0076-5)
Supplement: Supplementary file 1 — Additional file 1. Code for the distributions. [file 12962_2017_76_MOESM1_ESM.docx]

**how the parameter for gamma distribution were computed**

///A shape (r) of 5 was selected so that distribution of skewed

//The value of lambda as computed from the r divided by the point estimate of //the costs.

costPHCsalaries 3450

costinpatientdies 49420

costinpatientlives 123550

costRDT 545

**how the parameter for beta distribution were computed**

Alpha the shape parameter was set at 2 to allow for a large variance.

Beta (the second shape parameter) was computed from the mode and alpha using the following equation (Paul Johnson and Matt Beverlin, page 10):

**rcode for developing the random variables the excel document for details**

costPHCsalaries<-rgamma(n, 5, 0.001449275)

costinpatientdies<-rgamma(n,5, 0.000101174)

costinpatientlives<-rgamma(n, 5, 0.0000404694)

totalyldnotadmittedsurvived<-rgamma(n,5,0.148441204)

totalyldadmittedalive<-rgamma(n,5, 0.149237694)

totalyldadmitteddied<-rgamma(n,5, 2.883451831)

pNoRectal<-rbeta(n,2, 2)

pCompliance<-rbeta(n,2, 1.490312966)

costRDT<- rgamma(n, 5, 0.009174312)

pEIRectal<-rbeta(n, 2, 2.040816327)

pinpatientCFR<-rbeta(n, 13.33333333)
