# Supplementary material for: Cost effectiveness of pre-referral antimalarial treatment in severe malaria among children in sub-Saharan Africa
Source: Cost Eff Resour Alloc. 2017 Jul 14;15:14. doi: 10.1186/s12962-017-0076-5 (PMC5512821; doi:10.1186/s12962-017-0076-5)
Supplement: Supplementary file 6 — Additional file 6. Results for the base model. [file 12962_2017_76_MOESM6_ESM.pdf]

PRIMARY HEALTH CARE WORKERS

```
>PHCFdaly
        [,1]
[1,] 7512.089
>PHCFcostemv
        [,1]
[1,] 88961.36
>PHCFdalysaverted
        [,1]
[1,] 12610.69
>PHCFincrementalcost
        [,1]
[1,] 88960.47
>PHCFicer
        [,1]
[1,] 7.054373
>
```

> ##summary CHW

```
>CHWdaly
       [,1]
[1,] 4587.76
>CHWcostemv
        [,1]
[1,] 85491.71
>CHWdalysaverted
        [,1]
[1,] 15535.01
>CHWincrementalcost
        [,1]
[1,] 85490.82
>CHWicer
        [,1]
[1,] 5.503105
>
```

> ####TERTIARY HEALTH – SUMMARY MEASURES

```
>
>THFdalysaverted<-( notreatmentdaly- THFdaly)
>THFincrementalcost<-(THFcostemv-notreatmentcostemv)
>THFcostperDALYaverted= THFcostemv/THFdalysaverted
>THFicer= THFincrementalcost /THFdalysaverted
>THFdaly
       [,1]
[1,] 2186.05
>THFcostemv
        [,1]
[1,] 123711.6
```

```
>THFdalysaverted
        [,1]
[1,] 17936.72
>THFincrementalcost
        [,1]
[1,] 123710.8
>THFicer
        [,1]
[1,] 6.897065
>
```

NO TREATMENT OPTION

```
>notreatmentdaly
        [,1]
[1,] 20122.77
>notreatmentcostemv
         [,1]
[1,] 0.8882892
>
```
